# Formation of Liver Metastases Is Accompanied by Accelerated Musculoskeletal Deficits in LLC Tumor Hosts

**DOI:** 10.3390/ijms27104426

**Published:** 2026-05-15

**Authors:** Paola Ortiz Gonzalez, Anna M. Miller, Luis F. Cardona Polo, Lilian I. Plotkin, Fabrizio Pin, Joshua R. Huot

**Affiliations:** 1 Indiana Center for Musculoskeletal Health, Indiana University School of Medicine, Indianapolis, IN 46202, USAlplotkin@iu.edu (L.I.P.);; 2 Department of Anatomy, Cell Biology and Physiology, Indiana University School of Medicine, Indianapolis, IN 46202, USAlfcardon@iu.edu (L.F.C.P.); 3 Roudebush Veterans Administration Medical Center, Indianapolis, IN 46202, USA; 4 Simon Comprehensive Cancer Center, Indiana University School of Medicine, Indianapolis, IN 46202, USA

**Keywords:** musculoskeletal health, cachexia, lung cancer, muscle, bone

## Abstract

Lung cancer is a leading cause of death worldwide and is often accompanied by declines in musculoskeletal health (i.e., cachexia). Despite affecting a majority of lung cancer patients, cachexia remains understudied and currently has no cure. We have previously demonstrated that liver metastases (LMs) exacerbate cachexia in murine models of colorectal cancer, and, while the liver represents a common site of metastases and is associated with poor prognosis in patients with lung cancer, whether LMs heighten musculoskeletal wasting in mice bearing lung cancer is unknown. Here, we aimed to characterize the impact of LMs on musculoskeletal health in a mouse model of lung cancer cachexia. C57BL/6J male mice were injected with LLC tumor cells either subcutaneously or intrasplenically (LMs) to mimic hepatic metastases (*n* = 6–9/group). Upon sacrifice, skeletal muscle, bone, and plasma were collected for morphological and molecular analyses. Consistently, compared to healthy controls, metastatic tumor hosts displayed greater reductions in muscle weights (~17%), in line with decreased muscle torque (~23%) and reduced muscle cross-sectional area (~10%). On a molecular level, skeletal muscle from mice bearing LMs had elevated levels of pStat3, Murf1, and Atrogin-1, suggesting enhanced protein catabolism. Similar to skeletal muscle, metastatic tumor hosts displayed greater losses in trabecular bone and increased skeletal fragility. Plasma proteomics identified 211 and 131 differentially expressed proteins in metastatic hosts compared to control animals and subcutaneous LLC hosts, respectively. Top regulated pathways in mice bearing LMs included neutrophil degranulation, BAG2 signaling, and cachexia signaling. Overall, our findings demonstrate that LMs are accompanied by accelerated musculoskeletal wasting and weakness in a mouse model of lung cancer cachexia. This work highlights the need for animal models that mimic advanced cancer, thus providing a better understanding of the mechanisms that mediate cachexia.

## 1. Introduction

By the end of 2026, it is estimated that more than 229,000 new cases of lung cancer will be diagnosed, remaining steadfast as one of the most prevalent cancers in the United States [1]. Further, lung cancer is among the least favorable in terms of prognosis, with a 5-year survival of 28% and nearly 125,000 deaths expected this year alone [1]. Despite declining rates in new cases, lung cancer remains the leading cause of cancer-related death in the United States and is 2.5 times higher than colorectal and pancreatic cancer [1]. Notably, it is estimated that 40–50% of lung cancer patients will develop cachexia, a debilitating syndrome characterized by musculoskeletal wasting and weakness. Known as a progressive condition, the development of cachexia heightens treatment toxicities, impairs the ability to perform daily activities of living, worsens overall survival, and is ultimately responsible for up to 30% of cancer-related deaths [2,3,4,5,6,7]. Despite its negative consequences on quality of life, effective treatments to counteract cachexia remain elusive.

One factor limiting progress in combating cachexia is the availability of small animal models that reflect the clinical population. This is especially true regarding models of advanced, metastatic cancer, where the prevalence and degree of cachexia occur at higher rates. Recent efforts from our group and others have attempted to narrow this gap, utilizing murine models of advanced cancer and, importantly, demonstrating that metastatic disease exacerbates skeletal muscle wasting and weakness [8,9,10]. Furthermore, use of these models has demonstrated that advanced cancer, in particular liver metastases (LMs) promotes system-wide wasting beyond that seen in routinely employed subcutaneous allograft and xenograft approaches. However, these studies have modeled advanced colorectal cancer, and despite the increased prevalence of cachexia in metastatic lung cancer patients [11], preclinical models of advanced lung cancer cachexia are lacking.

Using the commonly used Lewis lung carcinoma (LLC) cell line, in the present study we examined the impact of lung LMs on the development of cachexia. Here, we demonstrated that the formation of LLC LMs is accompanied by accelerated muscle wasting, muscle weakness, and bone fragility. Moreover, metastatic tumor hosts displayed systemic protein alterations distinct from non-metastatic tumors, providing insight into how advanced cancer may exacerbate cachexia. Together, our study supports further use of preclinical models that reflect the population of advanced cancer patients.

## 2. Results

### 2.1. LLC Liver Metastases Are Accompanied by Accelerated Body Weight Loss, Muscle Wasting, and Weakness

**Figure 1:**
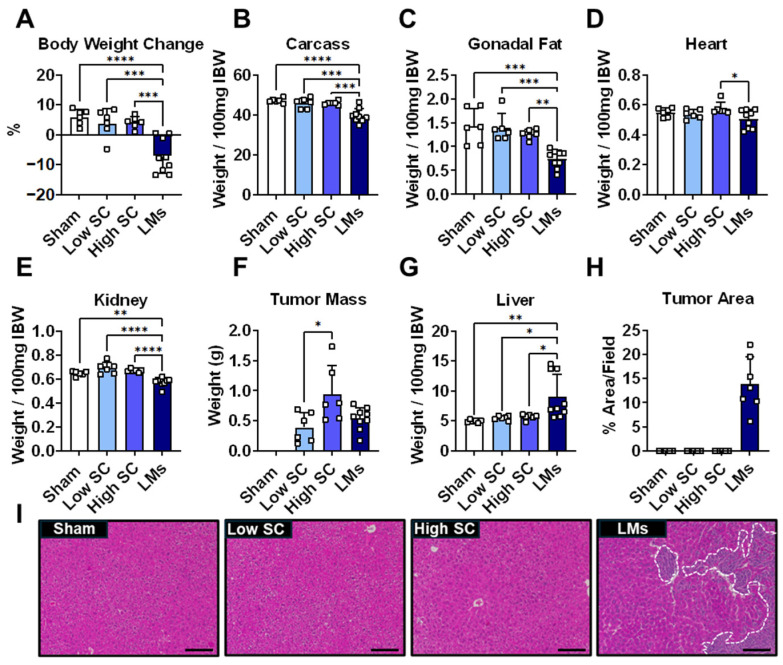
Accelerated weight loss in metastatic LLC tumor hosts. (**A**) Body weight change (tumor-free), (**B**) carcass, (**C**) gonadal fat, (**D**) heart, (**E**) and kidney weights normalized to initial body weight (IBW); (**F**) tumor mass, (**G**) liver weight (relative to IBW), (**H**) quantification of tumor area and representative (**I**) H&E staining of livers from 8-week-old C57BL6 male mice injected with saline (sham) or LLC tumor cells (*n* = 6–9 per group: panels (**A**–**G**); *n* = 5–7 per group: panels (**H**,**I**)). Tumor groups: 1.25 × 10^5^ subcutaneous (low SC); 1 × 10^6^ subcutaneous (high SC); 1.25 × 10^5^ intrasplenic (LMs). Representative images of liver H&E staining were taken at 10× magnification. Significant differences: * *p* < 0.05, ** *p* < 0.01, *** *p* < 0.001, and **** *p* < 0.0001.

**Figure 2:**
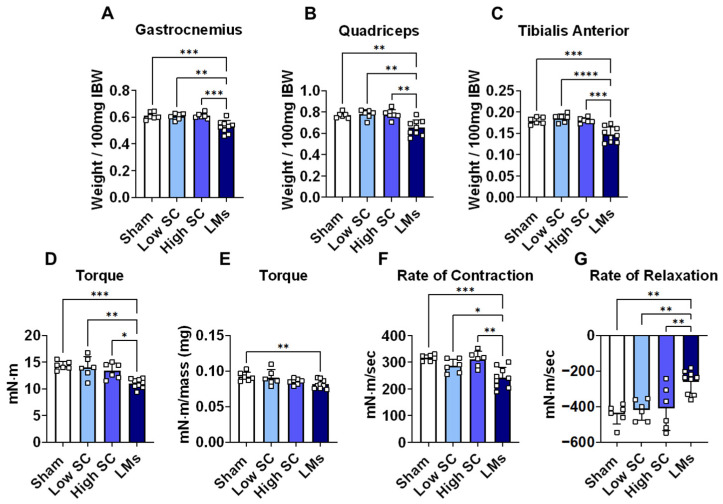
Metastatic LLC tumor hosts display accelerated muscle wasting and weakness. (**A**) Gastrocnemius, (**B**) quadriceps, and (**C**) tibialis anterior weights normalized to initial body weight (IBW); (**D**) plantarflexion torque assessed at 125 Hz (expressed as millinewton-meters); (**E**) torque normalized to weight of gastrocnemius muscle; (**F**) maximum rate of contraction and (**G**) maximum rate of relaxation (expressed in millinewton-meters per second). Measures were taken in 8-week-old C57BL6 male mice injected with saline (sham) or LLC tumor cells (*n* = 6–9 per group). Tumor groups: 1.25 × 10^5^ subcutaneous (low SC); 1 × 10^6^ subcutaneous (high SC); 1.25 × 10^5^ intrasplenic (LMs). Significant differences: * *p* < 0.05, ** *p* < 0.01, *** *p* < 0.001, and **** *p* < 0.0001.

We have previously demonstrated that LMs exacerbate cachexia in mouse models of colorectal cancer [8,9,10] and thus wanted to assess whether formation of lung LMs would also promote a heightened cachectic phenotype. Male C57BL/6 mice were injected with LLC cells intrasplenically to promote LMs, or subcutaneously (low SC and high SC), which is routinely employed to study cachexia. Mice with LLC LMs displayed significant weight loss 18 days following tumor cell injection, which did not occur in either SC group (Figure 1A). In addition, SC tumor mice did not display phenotypic indices of cachexia at 18 days, reflected by unchanged carcass weight and fat mass. In contrast, animals with LMs displayed lower carcass, gonadal fat, and kidney weights compared to all groups (Figure 1B–E), suggesting accelerated development of body wasting. Liver mass was increased in the LMs group, as expected given the development of metastases, while primary tumor mass was elevated in high SC compared to other LLC groups (Figure 1F–I). Much like body weight, carcass weight, fat mass, and skeletal muscle mass were unchanged at 18 days in SC tumor hosts compared to sham (Figure 2A–C). In a separate experiment, mice were injected with LLC cells (SC; high) and euthanized after 25 days, yielding increased primary tumor mass compared to all experimental groups at 18 days (Supplementary Figure S1A). While body weight and gondal fat were reduced in SC tumor hosts at 25 days, reductions in both parameters were greater in mice bearing LMs at 18 days (Supplementary Figure S1B,C). In further support of accelerated cachexia, LM hosts displayed lower gastrocnemius, quadriceps, and tibialis anterior muscle weights compared to sham (−17% on average), low SC (−18% on average), and high SC (−17% on average) at 18 days (Figure 2A–C). In addition, LM hosts displayed greater reductions in muscle weights (−8% on average) compared to SC hosts at 25 days (Supplementary Figure S1D–F). Moreover, LM hosts displayed impaired muscle contractility, reflected by reductions in absolute plantarflexion torque (−23.3% vs. sham; −17.8% vs. high SC; −21.6% vs. low SC), normalized torque (−11.7% vs. sham), max rate of contraction (−23.5% vs. sham; −22.2% vs. high SC; −15.3% vs. low SC), and max rate of relaxation (−40.9% vs. sham; −36.5% vs. high SC; −37.7% vs. low SC) compared to all other groups at 18 days (Figure 2D–G). Together, these data indicate that LLC LM are accompanied by accelerated cachexia in tumor hosts.

### 2.2. Heightened Catabolic Signaling in Skeletal Muscle of Metastatic LLC Tumor Hosts

**Figure 3:**
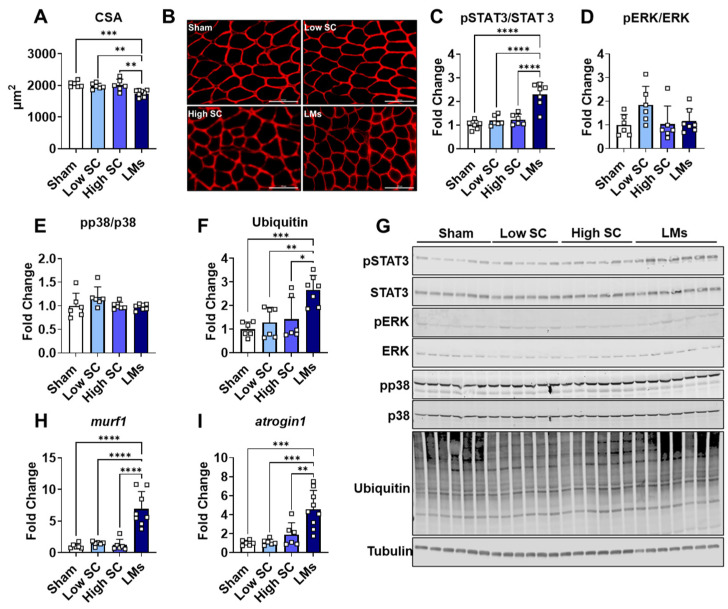
Heightened catabolic signaling in mice bearing LLC liver metastases. (**A**) Cross-sectional area and (**B**) representative dystrophin staining (20×) in 8-week-old C57BL6 male mice injected with saline (sham) or LLC tumor cells (*n* = 6–9 per group). (**C**–**G**) Quantification and representative western blotting of skeletal muscle phosphorylated (p)STAT3, STAT3, pERK, ERK, pp38, p38, Ubiquitin, and tubulin. Ubiquitin was normalized to tubulin and phosphorylated proteins were normalized to total proteins. (**H**,**I**) Skeletal muscle gene expression of *murf1* and *atrogin1.* Tumor groups: 1.25 × 10^5^ subcutaneous (low SC); 1 × 10^6^ subcutaneous (high SC); 1.25 × 10^5^ intrasplenic (LMs). Significant differences: * *p* < 0.05, ** *p* < 0.01, *** *p* < 0.001, and **** *p* < 0.0001.

Given the observations of accelerated wasting in metastatic LLC hosts, we wanted to assess whether LMs were also accompanied by greater fiber atrophy. Due to differences in timing, experimental animals euthanized at 18 days were used for all subsequent measures. Complementing reductions in skeletal muscle mass, fiber cross-sectional area was reduced in mice bearing LLC LMs compared to all groups (−14.4% vs. sham; −13.4% vs. high SC; −11.8% vs. low SC), while no atrophy was observed in either SC tumor group (Figure 3A,B). Molecular indices of heightened protein catabolism, often characteristic of cachectic muscle, were also assessed. In line with prior findings [8], STAT3 phosphorylation was markedly increased (>2-fold) in mice bearing LMs compared to sham, low SC, and high SC groups (Figure 3C,G). Despite unchanged signaling in ERK or p38, hosts with LLC LMs displayed greater ubiquitination of skeletal muscle protein, along with elevated gene expression of E3 ligases, *murf1* and *atrogin1* (Figure 3D–I). Overall, our findings suggest heightened catabolism in skeletal muscle of metastatic LLC hosts.

### 2.3. Mice Bearing LLC Liver Metastases Exhibit Elevated Skeletal Turnover and Fragility

**Figure 4:**
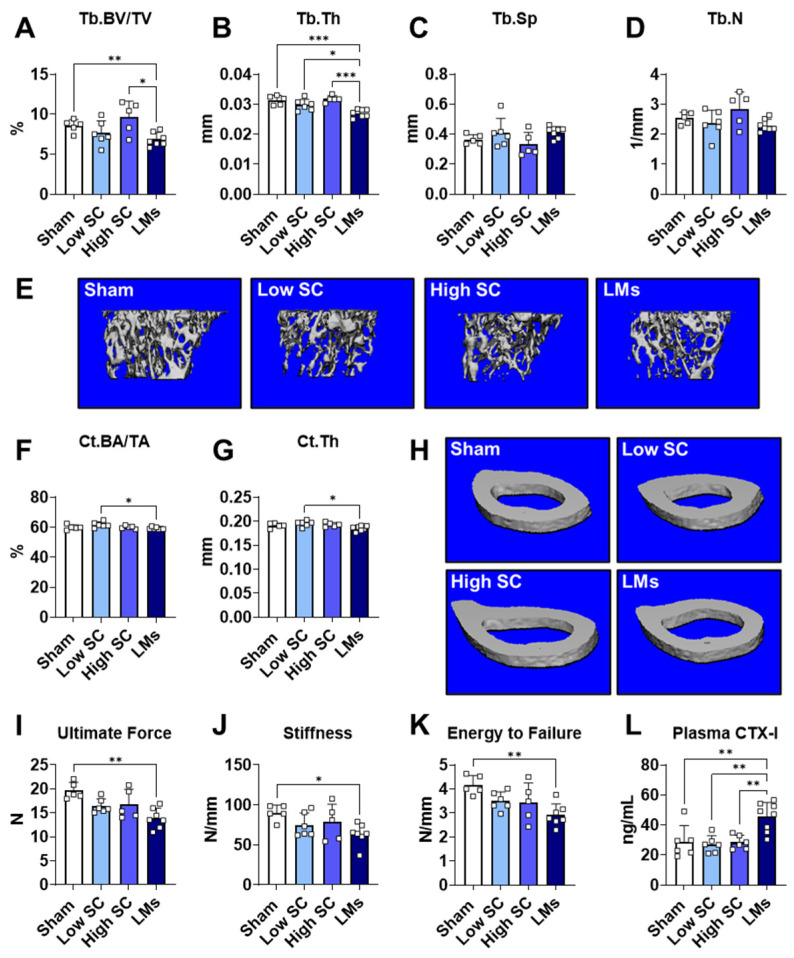
Mice bearing LLC liver metastases display elevated bone turnover and fragility. (**A**) Quantification of trabecular bone volume fraction (Tb.BV/TV), (**B**) trabecular thickness (Tb.Th), (**C**) trabecular separation (Tb.Sp), (**D**) trabecular number (Tb.N), and (**E**) representative three-dimensional microcomputed tomography (µCT) scanned images of humeri. (**F**) Quantification of cortical BA/TA (Ct.BA/TA), (**G**) cortical cross-sectional thickness (Ct.Th), and (**H**) representative cortical µCT images. Three-point bend testing to failure on femur bones; quantification of (**I**) ultimate force, (**J**) stiffness, and (**K**) energy to failure. (**L**) Plasma CTX-I levels assessed by ELISA. All measures were recorded from 8-week-old C57BL6 male mice injected with saline (sham) or LLC tumor cells (*n* = 6–9 per group). Tumor groups: 1.25 × 10^5^ subcutaneous (low SC); 1 × 10^6^ subcutaneous (high SC); 1.25 × 10^5^ intrasplenic (LMs). Significant differences: * *p* < 0.05, ** *p* < 0.01, and *** *p* < 0.001.

In addition to greater muscle wasting, prior work indicates that colorectal LMs may accelerate bone loss [8,9]. Thus, to investigate the impact of lung LMs on the skeleton, we assessed trabecular and cortical bone microarchitecture. Similar to other phenotype measures, SC tumor hosts displayed preserved trabecular bone compared to sham. Meanwhile, mice bearing LMs had lower Tb.BV/TV compared to sham (−19.7%) and high SC (−28.7%) as well as reduced Tb.Th compared to all groups (−14% vs. sham; −14.9% vs. high SC; and −9.2% vs. low SC; Figure 4A–E). For cortical measures, main effects were observed for Ct.BA/TA (*p* < 0.05) and Ct.Th (*p* < 0.05); however, hosts with LMs were only significantly reduced compared to low SC (Ct.BA/TA: −3.9%; Ct.Th: −5.1%; Figure 4F–H). Despite the limited change in cortical bone, biomechanical testing via 3-point bending to failure revealed marked bone fragility in mice bearing LLC LMs. While neither SC group displayed significant reductions in any biomechanical parameter, mice with LMs had reduced ultimate force (−29.4%), stiffness (−29.5%), and energy to failure (−30.1%) compared to sham (Figure 4I–K). Lastly, confirming heightened bone resorption, mice bearing LLC LMs displayed elevated plasma CTX-I levels (+58.5% vs. sham; +59.8% vs. high SC; +68.1% vs. low SC) compared to all other groups (Figure 4L). Together these results suggest that lung LMs induce trabecular bone loss and compromise bone composition and resistance to mechanical failure, even in the absence of widespread architectural cortical deterioration.

### 2.4. Liver Metastases Are Associated with Systemic Alterations in LLC Tumor Hosts

**Figure 5:**
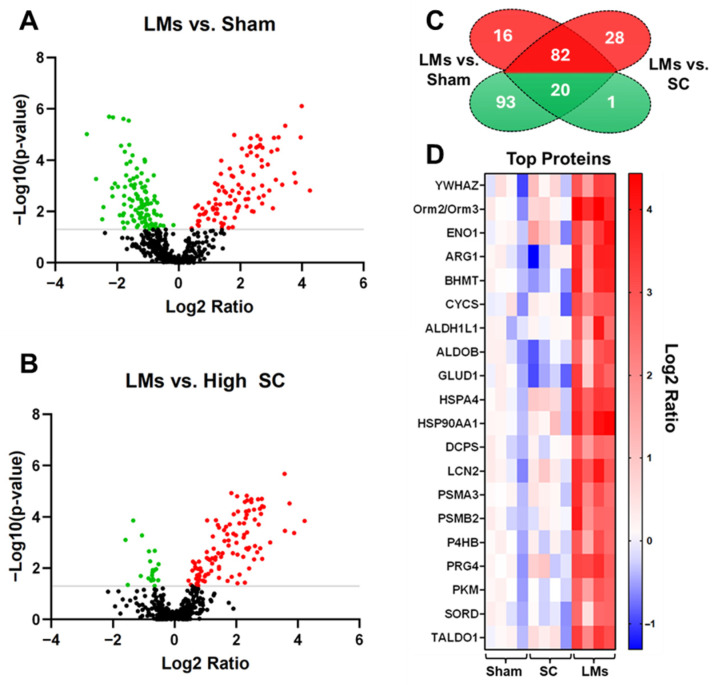
Liver metastases alter the plasma proteome in LLC tumor hosts. Volcano plots of differentially expressed plasma proteins in mice bearing LLC LMs vs. (**A**) sham and (**B**) subcutaneous (SC) tumor hosts (upregulated proteins are marked red; downregulaged proteins are marked green). (**C**) Venn diagram of differentially expressed proteins, including the number of commonly elevated and commonly reduced proteins in LLC LMs; (**D**) heatmap of top 20 altered proteins in LLC LMs tumor hosts. Analysis was conducted on 8-week-old C57BL6 male mice injected with saline (sham) or LLC tumor cells (*n* = 4 per group). Tumor groups: 1 × 10^6^ subcutaneous (high SC); 1.25 × 10^5^ intrasplenic (LMs). The cut-off was set at *p* < 0.05.

**Figure 6:**
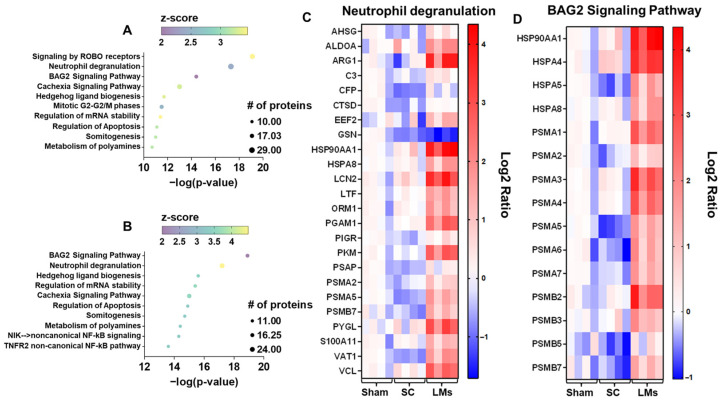
Upregulated pathways in mice bearing LLC liver metastases. Top 10 altered pathways in LMs vs. (**A**) sham and vs. (**B**) high SC. Pathways are plotted based on −log(*p*-value); the size of the dot corresponds to the number of proteins within a given pathway; the z-score value is reflected by color. Log2 ratio heatmap of proteins related to (**C**) neutrophil degranulation and (**D**) BAG2 signaling. Blue indicates reduction, and red indicates elevation of the protein.

To gain insight into the systemic changes that may be responsible for heightened cachexia in LLC LMs, proteomics was performed on plasma from the following experimental groups: sham, high SC, and LMs. LM hosts exhibited differential abundance (cut-off: *p* < 0.05) of 211 proteins (113 proteins decreased and 98 proteins increased) compared to sham and 131 proteins (21 proteins decreased and 110 proteins increased) compared to SC hosts. Interestingly, there was substantial overlap in the altered proteins when comparing LMs to sham and to SC hosts, with 82 increased proteins and 20 decreased proteins in common (Figure 5A–D). The elevated differentially abundant proteins were then used to run biological process and pathway analyses. Given the overlap of increased proteins in LMs vs. sham and LMs vs. high SC, it is unsurprising that several top pathways were also in concert, including neutrophil degranulation, BAG2 signaling, cachexia signaling, hedgehog ligand biogenesis, and regulation of apoptosis (Figure 6A–D).

**Figure 7:**
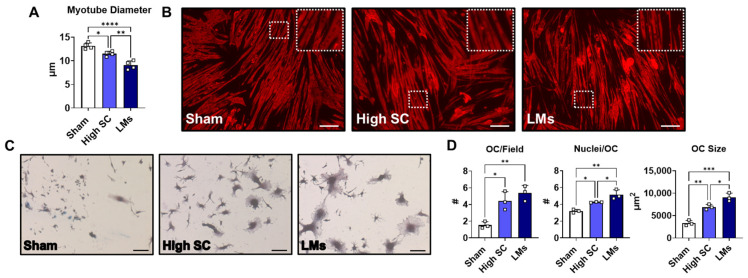
Plasma from metastatic LLC promotes musculoskeletal alterations in vitro. (**A**) Quantification of diameter and (**B**) representative images of C2C12 myotubes (5×; enlarged portion within white box) exposed to plasma from sham, high SC and LMs. (**C**) Representative images (10×) and (**D**) quantification of osteoclast (OC) differentiation of RAW264.7 cells following exposure to plasma-conditioned media from sham, high SC, and LMs. OC parameters measured included number per field, nuclei per OC, and size. Significant differences: * *p* < 0.05, ** *p* < 0.01, *** *p* < 0.001, and **** *p* < 0.0001.

We then wanted to clarify whether systemic changes observed in LM hosts were sufficient to induce musculoskeletal alterations in vitro. Differentiated C2C12 myotubes were exposed to media supplemented with plasma (2%) from sham, high SC, and LMs for 48 h and assessed for myotube atrophy. Plasma from high SC tumor hosts was sufficient to induce myotube wasting (−12.6%) compared to sham-treated myotubes (Figure 7A,B). In line with muscle wasting observed in metastatic tumor hosts, C2C12s exposed to plasma from LMs exacerbated wasting relative to sham (−31%) and were significantly lower than high SC-treated myotubes (−21.1%). In separate experiments, C2C12 myotubes were exposed to conditioned media generated from AML12-LLC co-cultures: either transwell (tLLC) or mixed to mimic metastases (mLLC). C2C12s exposed to conditioned media from tLLC were atrophic (−29.4%) compared to the control. Similar to the plasma-treated C2C12s, mLLC-conditioned media exacerbated atrophy, reducing myotube diameter compared to both control (−44.9%) and tLLC (−22%; Supplementary Figure S2). Next, RAW264.7 cells were cultured in plasma (1%) along with RANKL for 4 days and assessed for osteoclast formation. Plasma from high SC was sufficient to increase osteoclast number (+189%), nuclei per osteoclast (+33%), and osteoclast size (+108%) compared to sham (Figure 7C,D). Similar to the evidence of greater bone turnover in metastatic tumor hosts, plasma from LMs further elevated osteoclast formation, yielding increases in nuclei per osteoclast (+173% vs. sham; +31% vs. high SC) and osteoclast size (+61% vs. sham; +21% vs. high SC) compared to both sham and high SC.

## 3. Discussion

Despite advances in treatment and steep declines in cigarette smoking, lung cancer remains the leading cause of cancer-related mortality [1]. Alarmingly, incidence rates among younger populations and nonsmokers have been increasing, signaling that lung cancer will persist as a major health challenge for years to come [12,13]. Compounding this burden is the high prevalence of cancer cachexia, a debilitating wasting syndrome that severely diminishes quality of life and accounts for up to 30% of cancer deaths [2,3,4,5,6,7]. While cachexia has traditionally been defined by progressive skeletal muscle loss, emerging evidence indicates that it encompasses widespread metabolic and physiological disruptions, necessitating a paradigm shift in how this condition is studied. This is particularly true in lung cancer patients and preclinical modeling of advanced colorectal cancer, where the prevalence and degree of cachexia are elevated [8,9,10,11]. Given the lack of viable countermeasures against cachexia, in the present study we have attempted to increase the preclinical modeling toolkit, in particular for lung cancer cachexia.

The present study is an extension of prior work, demonstrating that metastatic colorectal cancer exacerbates cachexia and promotes differential signaling within skeletal muscle [8,9,10]. While not as common as in colorectal cancer, metastatic spread to the liver is known to be a strong independent predictor of survival in lung cancer patients [14,15,16]. Similar to previous findings in mouse models of colorectal cancer, LMs were seemingly accompanied by exacerbated cachexia in LLC tumor hosts. Indeed, mice bearing LLC LMs displayed greater body weight loss, fat wasting, muscle wasting, fiber atrophy, and muscle weakness compared to subcutaneous tumor hosts (Figure 1, Figure 2 and Figure 3). It is important to note that the primary experiment characterized in this study was only 18 days, which is shorter than the 28-day timeline often used in subcutaneous LLC cachexia studies [17,18]. The shortened timeline was in response to the weight loss (i.e., cachexia) and declining welfare of mice bearing LLC LMs. To make time-matched comparisons, all animals were euthanized at the same timepoint, prior to the development of cachexia in subcutaneous hosts. Nevertheless, these findings suggest that LMs are accompanied by rapid development of cachexia in LLC tumor hosts, reflecting a more advanced disease state. To try and account for the shortened timeframe of our initial experiment, a separate study with subcutaneous tumor hosts was extended to 25 days, which was sufficient to promote reductions in body weight, gonadal fat, and muscle mass (Supplementary Figure S1). Of interest, and despite the prolonged timing, the wasting observed at 25 days in SC tumor hosts did not reach the level of wasting observed at 18 days in mice bearing LMs. Taken together, the current data suggest that modeling advanced, metastatic lung cancer is accompanied by an accelerated cachexia phenotype.

Our findings reinforce the growing body of evidence implicating STAT3 as a key prognosticator of cancer-induced muscle wasting. Consistent with prior reports across multiple preclinical tumor models, including colorectal cancer (C26; Apc^min/+^), melanoma (B16), and ovarian cancer (ES-2) [19,20,21,22,23,24], we observed a marked increase in STAT3 phosphorylation in mice bearing LLC LMs (Figure 3). Moreover, elevated STAT3 coincided with enhanced protein degradation, as reflected by increased ubiquitination and upregulation of E3 ubiquitin ligases *atrogin1* and *murf1*, all of which have been consistently associated with muscle wasting [21,25,26,27,28]. Interestingly, not all signaling pathways previously linked to cachexia were altered in our metastatic LLC hosts. ERK phosphorylation reported to increase in C26 allograft-bearing mice [29] was unchanged in our model, along with no observed elevation in phosphorylated p38. Collectively, these data identify STAT3 as a possible mediator of muscle wasting in advanced LLC tumor hosts. However, additional studies are needed to confirm the STAT3-dependent effects on skeletal muscle in this model.

An emerging area of cancer cachexia research is the recognition that this morose syndrome involves systemic, multi-organ dysfunction. Our findings, along with previous reports, support the concept that bone loss frequently accompanies muscle wasting and that disruption of the bone–muscle axis may exacerbate disease progression [9,30,31,32]. Along these lines, formation of LLC LMs appeared to accelerate bone loss and fragility, evidenced by reductions in trabecular bone, greater plasma CTX-I and altered mechanical properties compared to healthy animals (Figure 4). The present findings are in concert with prior work demonstrating that colorectal LMs (C26) drove greater muscle wasting and bone loss compared to subcutaneous tumor hosts [9,30]. As mentioned above, the shortened timeline (18 days) was insufficient for non-LMs tumor hosts to develop cachexia, including bone deterioration, which has previously been shown to occur in mice bearing subcutaneous LLC tumors [33]. Given the higher turnover rate of trabecular compared to cortical bone, the shortened experimental timeline is also likely responsible for the lack of cortical bone loss in LLC LMs hosts, which has been found in SC LLC tumor-bearing mice after 4 weeks [33]. This same work also demonstrated that cachectic SC LLC hosts have reduced osteoblasts in the trabecular bone, suggesting negative regulation of bone formation, in addition to elevated resorption [33]. Thus, while elevated CTX-I reflects higher bone resorption in LLC LM hosts, it is certainly possible that blunted bone formation is also contributing to the skeletal phenotype. Nevertheless, these findings support the complexity of cachexia-related musculoskeletal pathology and highlight the need for systematic investigation of metastatic burden and timing in bone–muscle crosstalk. Future clinical and preclinical research should prioritize longitudinal studies to define the temporal dynamics of bone and muscle wasting, identify biomarkers of bone–muscle axis disruption, and evaluate integrated interventions that mitigate multi-organ decline during cancer progression.

Given the accelerated cachexia observed in metastatic LLC hosts, we wanted to assess the impact of advanced disease on circulating protein levels, and whether any altered proteins may be responsible for accelerating cachexia. Of interest, most of the altered proteins in LLC LM hosts were similar when compared to healthy animals or mice bearing subcutaneous tumors. As outlined above and perhaps unsurprisingly, proteins involved in inflammatory and degradation processes were markedly elevated in mice bearing metastases. The observed elevation of serum amyloid A-1, Alpha-1-acid glycoprotein 1 and 2, and SERPINA3 in metastatic LLC hosts, none of which were elevated in SC hosts, is consistent with activation of the hepatic acute phase response and systemic inflammation that underlies cachexia [19]. The elevation of Lipocalin-2 (LCN2) and S100A8/A9 further reflects activation of neutrophil and myeloid cell programs, with LCN2 directly involved in appetite regulation and S100A8/A9 representing biomarkers that correlate with tissue wasting severity [34,35]. Meanwhile, increased levels of Leucine-rich α-2-glycoprotein—which has been shown to induce myotube atrophy—and ITIH family members support the presence of inflammatory vascular and extracellular matrix remodeling processes associated with cachexia progression [36,37]. Further, the enrichment of complement and proteolytic cascade components, including C4bp, suggests activation of innate immune pathways in metastatic LLC hosts, which are increasingly recognized as integral drivers of systemic wasting in cancer cachexia [38].

In addition to elevated inflammation, previous findings in murine metastatic CRC suggest that LMs may disrupt systemic and hepatic energy balance to a greater degree than non-metastatic tumors [9]. In particular, metabolic profiling suggested that hepatic CRC metastases reduced liver glycogen, drove gluconeogenesis, and increased Krebs cycle activity [9]. Here, mice bearing LLC LMs had robust elevations in glycogen phosphorylase, suggesting exacerbated breakdown of liver glycogen. Similarly, LLC LMs drove marked elevations in several glycolytic/gluconeogenic enzymes, including fructose-bisphosphate aldolase (A/B), alpha-enolase, and phosphoglycerate mutase 1, as well as L-lactate dehydrogenase, suggesting increased gluconeogenesis and lactate shuttling into the Cori cycle. Additionally, malate dehydrogenase and fumarate hydratase levels were markedly elevated in LLC LM hosts, pointing to elevated Krebs cycle activity, further demonstrating the disruption in systemic energy metabolism. Collectively, this protein signature reflects a host response in metastatic LLC tumor-bearing mice characterized by acute phase activation, myeloid-induced inflammation, and immune-metabolic dysfunction, all of which contribute to the development and progression of cancer cachexia.

Overall, our findings demonstrate that the formation of LMs induces and is accompanied by accelerated cancer cachexia in LLC tumor hosts. Although we identified distinct systemic protein changes in the plasma of tumor-bearing hosts with LMs, several of which are linked to cachexia, the present study did not incorporate broad-scale omic approaches to how these systemic changes may uniquely alter signaling networks within skeletal muscle. Additionally, the present work did not assess how metastases alter hepatic endocrine function or how such changes could contribute to skeletal muscle wasting, necessitating future mechanistic studies to identify mediators of cachexia in this context. This may be of particular importance given the recent work highlighting the role of liver-derived hepatokines in the development of cachexia [37]. In addition, metastatic tumor burden was assessed solely through histological analyses, which may represent a limitation by precluding direct comparisons with tumor size in non-metastatic lung cancer hosts. Another limitation related to translational relevance is the rapid development of cachexia in this model. While the study successfully investigated cachexia in the context of lung cancer–associated LMs, future work could employ lower tumor cell doses or alternative lung cancer models to extend disease progression. Furthermore, the current study did not assess whether chemotherapy administration further exacerbates muscle wasting, despite previous work from our laboratory and others showing that several anticancer agents can induce cachexia independently of their effects on tumor growth [31,39,40,41]. Finally, this study was conducted exclusively in male animals, precluding assessment of potential sex-specific responses to LMs. Given reported sexual dimorphism in other murine cancer models [42], future studies should examine the differential effects of LMs on skeletal muscle in males versus females.

In conclusion, our findings demonstrate that LLC tumor infiltration of the liver is accompanied by rapid development of cachexia. This work also demonstrates that cancer can drive both skeletal muscle wasting and bone loss, warranting comprehensive assessment of musculoskeletal health in future cachexia studies. Collectively, our findings support the inclusion of the LLC LMs tumor model within the experimental toolkit to advance our understanding of cancer-associated musculoskeletal deficits.

## 4. Materials and Methods

### 4.1. Animals

Animal experiments were approved by the Institutional Animal Care and Use Committee at Indiana University School of Medicine and followed the National Institutes of Health Guidelines for Use and Care of Laboratory Animals. For the LLC experiments, 8-week-old male C57BL/6J mice (The Jackson Laboratory, Bar Harbor, ME, USA) were group-housed (up to 5 per cage) and randomized into one of the following conditions: subcutaneous injection of sterile saline (100 µL) and isovolumetric intrasplenic injection of sterile saline (sham, *n* = 6); subcutaneous injection of LLC cells (1.25 × 10^5^) in sterile saline (100 µL) and isovolumetric intrasplenic injection of sterile saline (low SC, *n* = 6); subcutaneous injection of LLC cells (1.00 × 10^6^) in sterile saline (100 µL) and isovolumetric intrasplenic injection of sterile saline (high SC, *n* = 6); subcutaneous injection of sterile saline (100 µL) and isovolumetric intrasplenic injection of LLC cells (1.25 × 10^5^) in sterile saline (LMs, *n* = 9). The approach to promote liver metastases (LMs) of LLC tumor cells was performed similar to our previous work with colorectal cancer cells [8,9,10]. In a separate experiment, 8-week-old male mice (C57BL/6J; The Jackson Laboratory) were injected subcutaneously with LLC cells (SC: 1.00 × 10^6^) or an equal volume of saline (sham, *n* = 5). At the time of euthanasia, blood (poor-platelet plasma), skeletal muscles (gastrocnemius, quadriceps, and tibialis anterior), bones (femurs and humeri), and other organs (heart, liver, gonadal fat, and kidneys) were harvested, weighed, and snap frozen.

### 4.2. Hematoxylin and Eosin Staining

To determine the formation of LLC LMs, liver tissue was fixed, paraffin embedded, and sectioned (7 μm) in preparation for hematoxylin and eosin staining as performed previously [43]. Hematoxylin and eosin-stained liver sections were then observed and imaged (5×) under an Axio Observer.Z1 motorized microscope (Zeiss, Oberchoken, Germany). The tumor area relative to the liver area (expressed as a percentage) was assessed using ImageJ 1.43 software.

### 4.3. In Vivo Muscle Contractility

Plantarflexion torque was assessed as previously described [44]. Briefly, the right hind foot and tibia were aligned at 90° and the foot was taped to a force transducer. The right knee was clamped at the femoral condyles, avoiding compression of the fibular nerve. To stimulate the tibial nerve, two disposable monopolar electrodes (Natus Neurology, Middleton, WI, USA) were placed subcutaneously, posterior/medial to the knee. Maximum twitch was first determined using supramaximal stimulations (0.2 ms square wave pulse). Peak plantarflexion torque was then assessed following a supramaximal square wave stimulation (0.2 ms) delivered at 125 Hz stimulation frequency. Maximum torque, rate of contraction and rate of relaxation were determined via the Dynamic Muscle Control/Data Acquisition and Dynamic Muscle Control Data Analysis programs (Aurora Scientific, Aurora, ON, Canada (version 5)). The gastrocnemius muscle, the primary plantarflexor was used to normalize torque values.

### 4.4. Assessment of Fiber Cross-Sectional Area

To determine skeletal muscle cross-sectional area, 10 μm cryosections were taken at the mid-belly of tibialis anterior muscles (CM1860 cryostat; Leica Biosystems, Nussloch, Germany) and processed for immunostaining following established protocols [43]. In brief, sections were blocked for 1 h at room temperature, then incubated overnight at 4 °C with a dystrophin primary antibody (1:50; #MANDRA11(8B11), Developmental Studies Hybridoma Bank, Iowa City, IA, USA). This was followed by a 1 h incubation at room temperature with a secondary antibody (AlexaFluor 555, 1:1000, A21127, Thermo Fisher Scientific, Waltham, MA, USA). Entire dystrophin-labeled sections were imaged and analyzed for cross-sectional area (CSA) using a Lionheart LX automated microscope (BioTek Instruments, Winooski, VT, USA).

### 4.5. Western Blotting

Protein extracts of skeletal muscle were prepared by homogenizing whole quadriceps in RIPA buffer (150 mM NaCl, 1.0% NP-40, 0.5% sodium deoxycholate, 0.1% SDS, and 50 mM Tris, pH 8.0) supplemented with protease (Roche, Indianapolis, IN, USA) and phosphatase (Thermo Scientific, Rockford, IL, USA) inhibitor cocktails on ice. After centrifugation (15 min at 14,000× *g*, 4 °C) to remove debris, protein concentrations were determined using a BCA assay kit (Thermo Scientific). Equal amounts of protein (25 μg) were separated on 4–15% gradient SDS Criterion TGX precast gels (Bio-Rad, Hercules, CA, USA) and transferred to nitrocellulose membranes (30 min at 100 V; Bio-Rad). Membranes were blocked for 1 h at room temperature with Intercept blocking buffer (LI-COR Biosciences, Lincoln, NE, USA) and incubated overnight at 4 °C with primary antibodies under gentle agitation. Following three PBST washes (PBS + 0.2% Tween-20), membranes were incubated for 1 h at room temperature with DyLight 800 anti-rabbit IgG or DyLight 680 anti-mouse IgG secondary antibodies (Cell Signaling Technologies, Danvers, MA, USA). After additional PBST washes, blots were visualized and quantified using an Odyssey CLx Imaging System (LI-COR Biosciences). Antibodies for phospho-STAT3 (#9145), STAT3 (#9139), phospho-ERK1/2 (#4370), ERK1/2 (#4695), phospho-p38 (#4511), p38 (#8690), and Ubiquitin (#58395) were obtained from Cell Signaling Technologies, while α-Tubulin (12G10), used as a loading control, was from the Developmental Studies Hybridoma Bank (Iowa City, IA, USA).

### 4.6. Real-Time Quantitative Polymerase Chain Reaction (qRT-PCR)

RNA was extracted from whole quadriceps using the miRNeasy Mini kit (Qiagen, Valencia, CA, USA), following the manufacturer’s provided guidelines. RNA was then quantified by using a Synergy H1 spectrophotometer (Biotek, Winooski, VT, USA). Total RNA was reverse transcribed into cDNA by using the Verso cDNA kit (Thermo Fisher Scientific). Transcript levels of trim63 (qMmuCIP0030132) and fbxo32 (qMmuCEP0054105; Bio-Rad) were determined by qRT-PCR (Cielo 6, Azure Biosystems, Dublin, CA, USA). Gene expression was normalized to actb (qMmuCEP0039589) levels using the standard 2^−ΔΔCT^ methods.

### 4.7. Bone Morphometry

After euthanasia, humeri were harvested, cleaned of soft tissue, and fixed in 4% neutral-buffered formalin until 7 days before scanning, during which they were stored in 70% ethanol. Micro-computed tomography (microCT) analysis was performed using a Scanco μCT 35 system (Scanco Medical AG, Brüttisellen, Switzerland) at an isotropic voxel size of 10 μm, 55 kVp, 120 μA, and 151 ms integration time. Cortical and trabecular bone parameters were evaluated at standardized sites: the cortical region was assessed at the mid-diaphysis over a 1 mm section, and trabecular bone was analyzed in a 1 mm region beginning 0.5 mm distal to the growth plate. Images were reconstructed and analyzed using Scanco software (Version 6.6). Trabecular morphometric parameters included trabecular volume fraction (Tb.BV/TV; %), thickness (Tb.Th; mm), separation (Tb.Sp; mm), and number (Tb.N; 1/mm). Cortical transverse morphometry was averaged within a mid-diaphyseal span of approximately 5% of humeral length to obtain measures of cortical volume fraction (Ct.BA/TA; %) and cortical thickness (Ct.Th; mm).

### 4.8. 3-Point Bend Testing

Biomechanical properties of the femora were evaluated using a 3-point bending test. Hydrated femora were positioned with the anterior surface facing up on a 7.5 mm span and loaded at a rate of 0.025 mm/s using an ElectroForce 5500 system (TA Instruments, New Castle, DE, USA) until failure. A pre-load of 1 N was applied by lowering the crosshead at a rate of 0.03 mm/s. The bones were then loaded at a crosshead displacement rate of 0.2 mm/s until the crosshead displacement reached 5 mm, at which point the test was terminated. The bones were positioned on two lower supports spaced 6 mm apart, with the load applied to the mid-diaphysis at a displacement rate of 0.03 mm/s until failure. Force-displacement data were collected, and mechanical properties (ultimate force, stiffness, and energy to failure) were derived from the generated curves.

### 4.9. Enzyme-Linked Immunosorbent Assay (ELISA)

Plasma samples were collected at the time of sacrifice via cardiac puncture and stored at −80 °C until analysis. Levels of C-terminal telopeptide of type I collagen (CTX-I) were measured using a RatLaps™ CTX-I ELISA kit (Immunodiagnostic Systems Inc., Gaithersburg, MD, USA), according to the manufacturer’s instructions. Concentrations were calculated from a standard curve and expressed as ng/mL.

### 4.10. Plasma Proteomics

Plasma samples (*n* = 4/group) were diluted in 8 M Urea, 100 mM Tris hydrochloride, pH 8.5. An estimated 50 µg of protein per sample was reduced with 5 mM tris (2-carboxyethyl) phosphine hydrochloride (TCEP, Sigma-Aldrich Cat No: C4706) for 30 min at room temperature to reduce the disulfide bonds and alkylated using 10 mM choloracetamide (CAA, Sigma Aldrich Cat No: C0267) for 30 min at RT, protected from light. Samples were diluted to 2 M Urea with 50 mM Tris pH 8.5, and proteolytic digestion was carried out with Trypsin/LysC Gold (0.5 µg, mass spectrometry grade, Promega Corporation Cat No: V5072, Madison, WI, USA) overnight at 35 °C. After digestion, samples were quenched with 0.4% trifluoroacetic acid (TFA, *v*/*v*, Fluka Cat No: 91699, Darmstadt, Germany) and desalted on a Waters 50 mg SepPak (WAT054955) with a wash of 1 mL of 0.5% FA followed by elution in three times 200 µL of 70% acetonitrile and 0.1% formic acid (FA). For TMTpro labeling, peptides were first resuspended in 100 mM triethylammonium bicarbonate (TEAB, pH 8.5, diluted from 1 M stock). Each sample was then labeled overnight at room temperature with 0.5 mg of Tandem Mass Tag Pro (TMTpro™) reagent (16-plex kit, manufacture’s instructions: Thermo Fisher Scientific, TMTpro™ Isobaric Label Reagent Set; Cat No: 44520, lot no. YL381333 and ZA381335; sham: 126, 127N, 129N, 129C; subcutaneous: 130N, 131N, 132C, 133N; liver mets: 133C, 134N, 134C, 135N). After confirming over 95% labeling efficiency, reactions were quenched with 0.3% hydroxylamine (*v*/*v*) at room temperature for 15 min. Labeled peptides were then mixed and dried by speed vacuum. Half of the combined sample was resuspended in 0.5% TFA and fractionated on a Waters Sep-Pak^®^ Vac cartridge (Waters™ Cat No: WAT054955, Milford, MA, USA) with a 1 mL wash of water, a 1 mL wash of 5% acetonitrile and 0.1% triethylamine (TEA), followed by elution in eight fractions of 12.5%, 15%, 17.5%, 20%, 22.5%, 25%, 30%, and 70% acetonitrile, all with 0.1% TEA). Mass spectrometry was then performed utilizing an EASY-nLC 1200 HPLC system (SCR: 014993, Thermo Fisher Scientific) coupled to an Exploris 480™ mass spectrometer with a FAIMSpro interface (Thermo Fisher Scientific). Each fraction was loaded onto an Aurora column (Ionopticks 25 cm, (Cat # AUR2-25075C18A) and run at 300 nl/min. (Thermo Fisher ES902) The gradient (Mobile phases A: 0.1% formic acid (FA), water; B: 0.1% FA, 80% acetonitrile (Thermo Fisher Scientific Cat No: LS122500)), was increased from 8–38% B over 98 min, 30–80% B over 10 min, held at 80% for 2 min, and dropped from 80–4% B over the final 5 min. The mass spectrometer was operated in positive ion mode, default charge state of 2, advanced peak determination on, and lock mass of 445.12003. Three FAIMS CVs were utilized (−45 CV, −55 CV, and −65 CV), each with a cycle time of 1 s and with identical MS and MS2 parameters. Precursor scans (m/z 375–1500) were done with an orbitrap resolution of 120,000, RF lens % 40, automatic maximum inject time, standard AGC target, minimum MS2 intensity threshold of 5 × 10^3^, and MIPS mode to peptide, including charges of 2 to 7 for fragmentation with 60 s dynamic exclusion. MS2 scans were performed with a quadrupole isolation window of 0.7 m/z, 34% HCD CE, 15,000 resolution, standard AGC target, automatic maximum IT, and fixed first mass of 100 m/z.

### 4.11. Mass Spectrometry Data Analysis

Resulting RAW files were analyzed in Proteome Discover™ 2.5 (Thermo Fisher Scientific) with a Mus musculus reference proteome (downloaded from UniProt (both reviewed and unreviewed sequences, downloaded 02/28/2023, 55,250 sequences plus common contaminants (71 sequences)). SEQUEST HT searches were conducted with full trypsin digest, a maximum number of 3 missed cleavages; a precursor mass tolerance of 10 ppm; and a fragment mass tolerance of 0.02 Da. Static modifications used for the search were (1) carbamidomethylation on cysteine (C) residues; (2) TMTpro label on N-termini of peptides; and (3) TMTpro label on lysine (K) residues. Dynamic modifications used for the search were oxidation of methionines, phosphorylation on serine, threonine, or tyrosine, methionine loss, or acetylation with methionine loss on protein N-termini. The Percolator False Discovery Rate was set to a strict setting of 0.01 and a relaxed setting of 0.05. IMP-ptm-RS node was used for all modification site localization scores. In the consensus workflows, peptides were not normalized or scaled. Values from both unique and razor peptides were used for quantification. Quantification methods utilized TMTpro isotopic impurity levels available from Thermo Fisher Scientific. Reporter ion quantification was allowed with an S/N threshold of 5 and a co-isolation threshold of 30%. Resulting grouped abundance values for each sample type, abundance ratio values, and respective *p*-values (ANOVA) from Proteome Discover were exported to Microsoft Excel. Differentially abundant proteins were identified at a *p*-value of 0.05. Protein heat maps were generated in GraphPad Prism (GraphPad Software 11.0.0, San Diego, CA, USA). Ingenuity pathway analysis (IPA; summer 2025 release) software was used to identify the top upregulated biological pathways.

### 4.12. In Vitro Experiments

To assess whether soluble factors from LLC LMs hosts had direct effects on the musculoskeletal system we performed a series of in vitro experiments. To determine effects on myofiber size, C2C12 myoblasts were grown in Dulbecco modified Eagle’s medium (DMEM, Gibco, NY, USA), supplemented with 10% heat-inactivated fetal bovine serum (FBS, Sigma-Aldrich, St. Louis, MO, USA), 100 U/mL penicillin, and 100 µg/mL streptomycin (pen/strep). At approximately 100% confluence, myoblasts were exposed to low serum DMEM (2% Horse Serum; 1% pen/strep) and differentiated for 5 days. Differentiated C2C12 myotubes were then treated with plasma-conditioned media for 48 h. Conditioned media were generated by supplementing DMEM (1% pen/strep) with 2% plasma from sham, high SC, and LMs mice. In a separate set of experiments, AML12 hepatocytes (American Type Culture Collection; Manassas, VA, USA) were grown to confluence in DMEM/F12 (10% FBS; 1% pen/strep; 10 µg/mL Insulin, 5.5 µg/mL Transferrin, 5 ng/mL Selenium, 40 ng/mL Dexamethasone) and then co-cultured with LLC cells using either permeable transwell inserts or directly (to mimic metastases) for 24 h. The media were changed and cells were incubated for an additional 24 h. Conditioned media were then collected and used to treat C2C12 myotubes (day 5) for 48 h. Following treatments, cells were fixed in ice-cold acetone–methanol (50:50) and incubated with an anti-myosin heavy chain antibody (MF-20, 1:100; Developmental Studies Hybridoma Bank, Iowa City, IA, USA) followed by Alexa Fluor 594-conjugated secondary antibodies (Invitrogen, Grand Island, NY, USA). Myotube size was quantified by measuring the average diameter of long, multinucleated fibers (300–400 fibers/well), excluding regions containing clustered nuclei, using calibrated images acquired with ImageJ (v1.43), as previously described [10]. To assess for changes in osteoclast differentiation, RAW 264.7 cells (ATCC) were plated in DMEM (10% FBS; 1% pen/strep) for 24 h and then cultured in alpha-minimal essential medium (α-MEM Gibco, Grand Island, NY, USA) with RANKL (20 ng/mL) and 1% plasma from sham, high SC, and LMs mice for four days. After four days, cultures were fixed with 4% paraformaldehyde for 10 min at room temperature and underwent TRAP staining in accordance with the manufacturer’s instructions (Sigma Aldrich, St. Louis, MO, USA). Wells were observed under light microscopy, and TRAP+ cells containing more than three nuclei were considered osteoclasts.

### 4.13. Statistics

All statistical analyses were performed using GraphPad Prism 11.0.0. A one-way ANOVA was used to compare phenotype and molecular parameters for the sham, low SC, high SC, and LMs groups. Two-way ANOVAs were implemented when comparing phenotype measures from day 18 (sham; LMs) and day 25 (sham; SC) groups. Multiple comparisons were made using Tukey’s post hoc test. Statistical significance was set at *p* ≤ 0.05, and the data are presented as means ± SD.

## Data Availability

All raw and processed mass spectrometry proteomics data have been uploaded to the MassIVE repository with accession MSV000101267 and cross-referenced with ProteomeXchange ID PXD076257.

## Supplementary Materials

The following supporting information can be downloaded at: https://www.mdpi.com/article/10.3390/ijms27104426/s1.

## References

[1] Siegel R.L., Kratzer T.B., Wagle N.S., Sung H., Jemal A. (2026). Cancer statistics, 2026. CA Cancer J. Clin..

[2] Dewys W.D., Begg C., Lavin P.T., Band P.R., Bennett J.M., Bertino J.R., Cohen M.H., Douglass H.O., Engstrom P.F., Ezdinli E.Z. (1980). Prognostic effect of weight loss prior to chemotherapy in cancer patients. Eastern Cooperative Oncology Group. Am. J. Med..

[3] Hayes S., Battistutta D., Newman B. (2005). Objective and subjective upper body function six months following diagnosis of breast cancer. Breast Cancer Res. Treat..

[4] Luctkar-Flude M., Groll D., Woodend K., Tranmer J. (2009). Fatigue and physical activity in older patients with cancer: A six-month follow-up study. Oncol. Nurs. Forum.

[5] Mustian K.M., Peppone L.J., Palesh O.G., Janelsins M.C., Mohile S.G., Purnell J.Q., Darling T.V. (2009). Exercise and Cancer-related Fatigue. US Oncol..

[6] Fearon K.C., Glass D.J., Guttridge D.C. (2012). Cancer cachexia: Mediators, signaling, and metabolic pathways. Cell Metab..

[7] Melstrom L.G., Melstrom K.A., Ding X.Z., Adrian T.E. (2007). Mechanisms of skeletal muscle degradation and its therapy in cancer cachexia. Histol. Histopathol..

[8] Huot J.R., Novinger L.J., Pin F., Bonetto A. (2020). HCT116 colorectal liver metastases exacerbate muscle wasting in a mouse model for the study of colorectal cancer cachexia. Dis. Model. Mech..

[9] Huot J.R., Novinger L.J., Pin F., Narasimhan A., Zimmers T.A., O’Connell T.M., Bonetto A. (2020). Formation of colorectal liver metastases induces musculoskeletal and metabolic abnormalities consistent with exacerbated cachexia. JCI Insight.

[10] Huot J.R., Pin F., Essex A.L., Bonetto A. (2021). MC38 Tumors Induce Musculoskeletal Defects in Colorectal Cancer. Int. J. Mol. Sci..

[11] Shiono M., Huang K., Downey R.J., Consul N., Villanueva N., Beck K., Fenn K., Dietz D., Yamaguchi T., Kato S. (2016). An analysis of the relationship between metastases and cachexia in lung cancer patients. Cancer Med..

[12] Pelosof L., Ahn C., Gao A., Horn L., Madrigales A., Cox J., McGavic D., Minna J.D., Gazdar A.F., Schiller J. (2017). Proportion of Never-Smoker Non-Small Cell Lung Cancer Patients at Three Diverse Institutions. J. Natl. Cancer Inst..

[13] LoPiccolo J., Gusev A., Christiani D.C., Janne P.A. (2024). Lung cancer in patients who have never smoked-An emerging disease. Nat. Rev. Clin. Oncol..

[14] Tseng S.E., Chiou Y.Y., Lee Y.C., Perng R.P., Jacqueline W.P., Chen Y.M. (2014). Number of liver metastatic nodules affects treatment options for pulmonary adenocarcinoma patients with liver metastases. Lung Cancer.

[15] Tang C., Liao Z., Hess K., Chance W.W., Zhuang Y., Jensen G., Xu T., Komaki R., Gomez D.R. (2016). Prognosis and predictors of site of first metastasis after definitive radiation therapy for non-small cell lung cancer. Acta Oncol..

[16] Kanaji N., Tadokoro A., Watanabe N., Inoue T., Kadowaki N., Ishii T. (2019). Association of specific metastatic organs with the prognosis and chemotherapeutic response in patients with advanced lung cancer. Respir. Investig..

[17] Morena da Silva F., Rosa-Caldwell M.E., Schrems E.R., Martinez L., Amos M.G., Lim S., Cabrera A.R., Brown J.L., Washington T.A., Greene N.P. (2022). PGC-1alpha overexpression is not sufficient to mitigate cancer cachexia in either male or female mice. Appl. Physiol. Nutr. Metab..

[18] Hain B.A., Xu H., VanCleave A.M., Gordon B.S., Kimball S.R., Waning D.L. (2021). REDD1 deletion attenuates cancer cachexia in mice. J. Appl. Physiol..

[19] Bonetto A., Aydogdu T., Kunzevitzky N., Guttridge D.C., Khuri S., Koniaris L.G., Zimmers T.A. (2011). STAT3 activation in skeletal muscle links muscle wasting and the acute phase response in cancer cachexia. PLoS ONE.

[20] Bonetto A., Aydogdu T., Jin X., Zhang Z., Zhan R., Puzis L., Koniaris L.G., Zimmers T.A. (2012). JAK/STAT3 pathway inhibition blocks skeletal muscle wasting downstream of IL-6 and in experimental cancer cachexia. Am. J. Physiol. Endocrinol. Metab..

[21] Pin F., Barreto R., Kitase Y., Mitra S., Erne C.E., Novinger L.J., Zimmers T.A., Couch M.E., Bonewald L.F., Bonetto A. (2018). Growth of ovarian cancer xenografts causes loss of muscle and bone mass: A new model for the study of cancer cachexia. J. Cachexia Sarcopenia Muscle.

[22] Baltgalvis K.A., Berger F.G., Pena M.M., Davis J.M., Muga S.J., Carson J.A. (2008). Interleukin-6 and cachexia in ApcMin/+ mice. Am. J. Physiol. Regul. Integr. Comp. Physiol..

[23] Narsale A.A., Enos R.T., Puppa M.J., Chatterjee S., Murphy E.A., Fayad R., Pena M.O., Durstine J.L., Carson J.A. (2015). Liver inflammation and metabolic signaling in ApcMin/+ mice: The role of cachexia progression. PLoS ONE.

[24] Pretto F., Ghilardi C., Moschetta M., Bassi A., Rovida A., Scarlato V., Talamini L., Fiordaliso F., Bisighini C., Damia G. (2014). Sunitinib prevents cachexia and prolongs survival of mice bearing renal cancer by restraining STAT3 and MuRF-1 activation in muscle. Oncotarget.

[25] Murphy K.T., Struk A., Malcontenti-Wilson C., Christophi C., Lynch G.S. (2013). Physiological characterization of a mouse model of cachexia in colorectal liver metastases. Am. J. Physiol. Regul. Integr. Comp. Physiol..

[26] Kwak K.S., Zhou X., Solomon V., Baracos V.E., Davis J., Bannon A.W., Boyle W.J., Lacey D.L., Han H.Q. (2004). Regulation of protein catabolism by muscle-specific and cytokine-inducible ubiquitin ligase E3alpha-II during cancer cachexia. Cancer Res..

[27] Milan G., Romanello V., Pescatore F., Armani A., Paik J.H., Frasson L., Seydel A., Zhao J., Abraham R., Goldberg A.L. (2015). Regulation of autophagy and the ubiquitin-proteasome system by the FoxO transcriptional network during muscle atrophy. Nat. Commun..

[28] Sandri M., Sandri C., Gilbert A., Skurk C., Calabria E., Picard A., Walsh K., Schiaffino S., Lecker S.H., Goldberg A.L. (2004). Foxo transcription factors induce the atrophy-related ubiquitin ligase atrogin-1 and cause skeletal muscle atrophy. Cell.

[29] Penna F., Costamagna D., Fanzani A., Bonelli G., Baccino F.M., Costelli P. (2010). Muscle wasting and impaired myogenesis in tumor bearing mice are prevented by ERK inhibition. PLoS ONE.

[30] Bonetto A., Kays J.K., Parker V.A., Matthews R.R., Barreto R., Puppa M.J., Kang K.S., Carson J.A., Guise T.A., Mohammad K.S. (2016). Differential Bone Loss in Mouse Models of Colon Cancer Cachexia. Front. Physiol..

[31] Essex A.L., Pin F., Huot J.R., Bonewald L.F., Plotkin L.I., Bonetto A. (2019). Bisphosphonate Treatment Ameliorates Chemotherapy-Induced Bone and Muscle Abnormalities in Young Mice. Front. Endocrinol..

[32] Hain B.A., Jude B., Xu H., Smuin D.M., Fox E.J., Elfar J.C., Waning D.L. (2019). Zoledronic acid improves muscle function in healthy mice treated with chemotherapy. J. Bone Miner. Res..

[33] Pin F., Prideaux M., Huot J.R., Essex A.L., Plotkin L.I., Bonetto A., Bonewald L.F. (2021). Non-bone metastatic cancers promote osteocyte-induced bone destruction. Cancer Lett..

[34] Olson B., Zhu X., Norgard M.A., Levasseur P.R., Butler J.T., Buenafe A., Burfeind K.G., Michaelis K.A., Pelz K.R., Mendez H. (2021). Lipocalin 2 mediates appetite suppression during pancreatic cancer cachexia. Nat. Commun..

[35] Liao W.C., Chen C.T., Tsai Y.S., Wang X.Y., Chang Y.T., Wu M.S., Chow L.P. (2023). S100A8, S100A9 and S100A8/A9 heterodimer as novel cachexigenic factors for pancreatic cancer-induced cachexia. BMC Cancer.

[36] Lee H., Kim A., Son K., Choi A., Cha S., Shin H., Kim N.S., Lee H. (2026). Comprehensive transcriptomic analysis identifies Lrg1 as a potential therapeutic target for preventing muscle atrophy in cancer cachexia. Am. J. Physiol. Cell Physiol..

[37] Kaltenecker D., Fisker Schmidt S., Weber P., Loft A., Morigny P., Machado J., Geppert J., Saul K.B., Benedikt P., Molocea C.E. (2025). Functional liver genomics identifies hepatokines promoting wasting in cancer cachexia. Cell.

[38] D’Lugos A.C., Ducharme J.B., Callaway C.S., Trevino J.G., Atkinson C., Judge S.M., Judge A.R. (2025). Complement pathway activation mediates pancreatic cancer-induced muscle wasting and pathological remodeling. J. Clin. Invest..

[39] Barreto R., Mandili G., Witzmann F.A., Novelli F., Zimmers T.A., Bonetto A. (2016). Cancer and Chemotherapy Contribute to Muscle Loss by Activating Common Signaling Pathways. Front. Physiol..

[40] Barreto R., Waning D.L., Gao H., Liu Y., Zimmers T.A., Bonetto A. (2016). Chemotherapy-related cachexia is associated with mitochondrial depletion and the activation of ERK1/2 and p38 MAPKs. Oncotarget.

[41] Huot J.R., Essex A.L., Gutierrez M., Barreto R., Wang M., Waning D.L., Plotkin L.I., Bonetto A. (2019). Chronic Treatment with Multi-Kinase Inhibitors Causes Differential Toxicities on Skeletal and Cardiac Muscles. Cancers.

[42] Zhong X., Zimmers T.A. (2020). Sex Differences in Cancer Cachexia. Curr. Osteoporos. Rep..

[43] Huot J.R., Pin F., Narasimhan A., Novinger L.J., Keith A.S., Zimmers T.A., Willis M.S., Bonetto A. (2020). ACVR2B antagonism as a countermeasure to multi-organ perturbations in metastatic colorectal cancer cachexia. J. Cachexia Sarcopenia Muscle.

[44] Huot J.R., Pin F., Bonetto A. (2021). Muscle weakness caused by cancer and chemotherapy is associated with loss of motor unit connectivity. Am. J. Cancer Res..

