# Supplementary material for: Formation of Liver Metastases Is Accompanied by Accelerated Musculoskeletal Deficits in LLC Tumor Hosts
Source: Int J Mol Sci. 2026 May 15;27(10):4426. doi: 10.3390/ijms27104426 (PMC13206901; doi:10.3390/ijms27104426)
Supplement: Supplementary file 1 [file ijms-27-04426-s001.zip › ijms-4259225-supplementary.pdf]

Supplementary

Formation of liver metastases is accompanied by accelerated musculoskeletal deficits in LLC tumor hosts

Paola Ortiz Gonzalez, Anna M. Miller, Felipe Cardona Polo, Lilian I. Plotkin, Fabrizio Pin, Joshua R. Huot

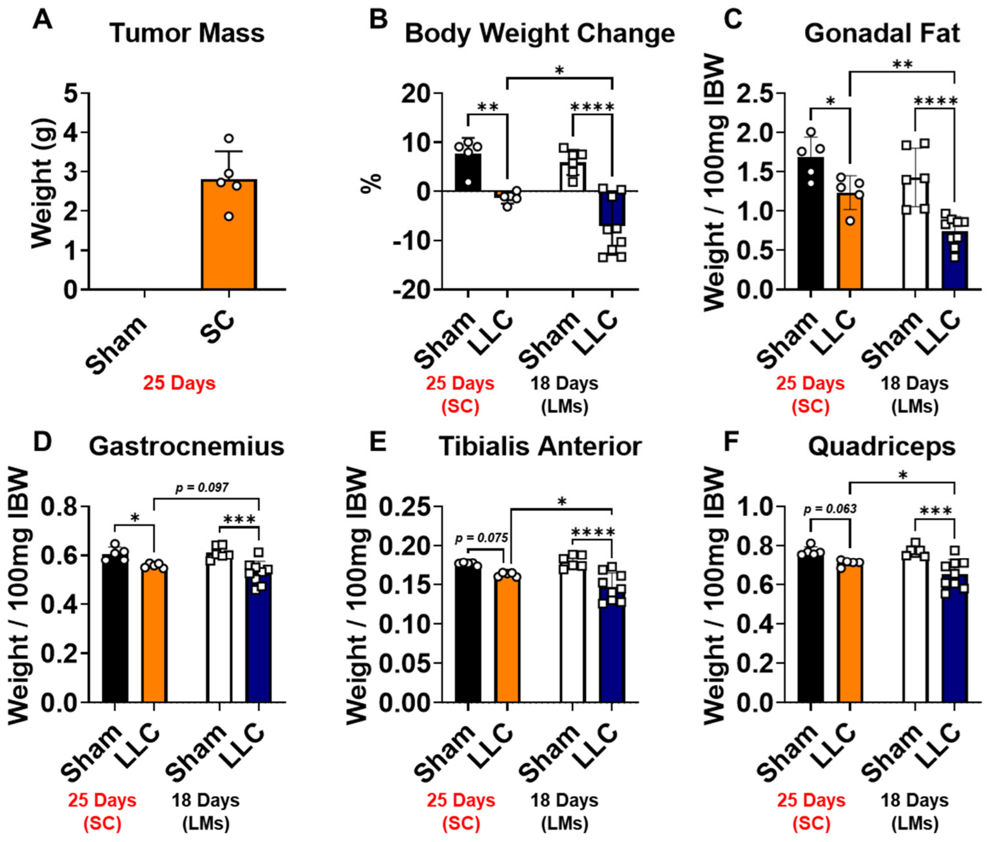

Figure S1. Mice bearing SC LLC tumors exhibit indices of cachexia 25 days post-implantation. (**A**) Tumor mass, (**B**) body weight change (tumor-free), (**C-F**) gonadal fat, gastrocnemius, tibialis anterior, and quadriceps, weights normalized to initial body weight (IBW) from 8-week-old C57BL6 male mice injected with saline (Sham) or LLC tumor cells (n=5-9 per group). Tumor groups: $1x10^6$ Subcutaneous (SC) euthanized at 25 days; $1.25x10^5$ intrasplenic (LMs) euthanized at 18 days. Significant differences: *p<0.05, **p<0.01, ***p<0.001, ****p<0.0001.

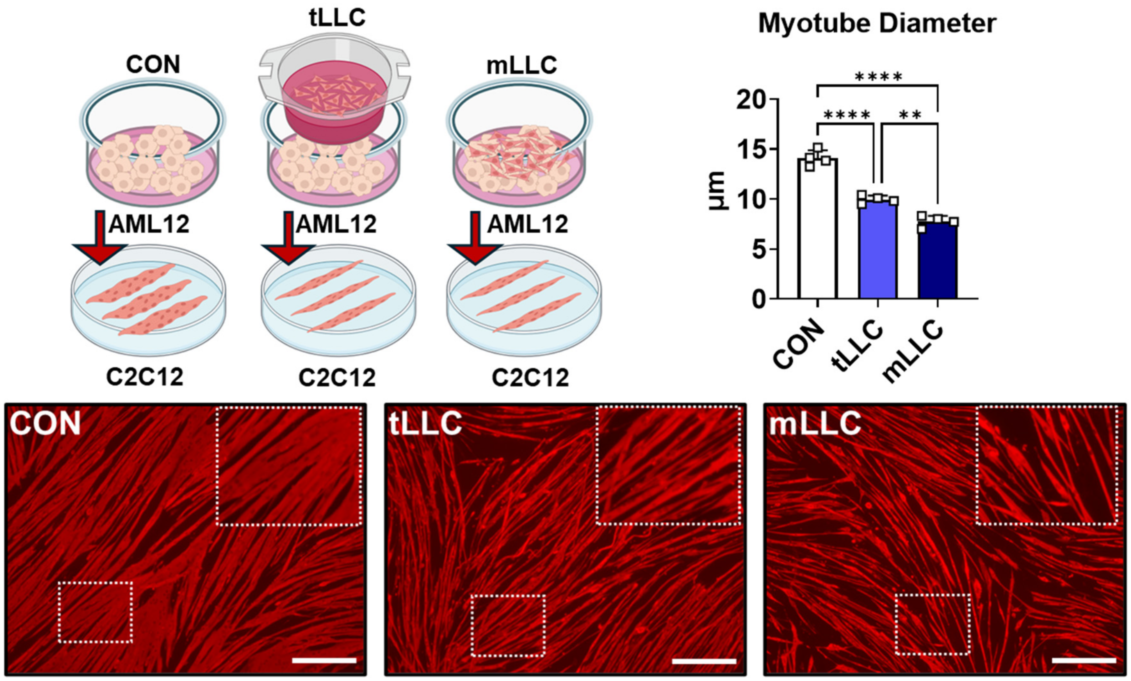

Figure S2. Mixed AML12-LLC conditioned media exacerbates myotube atrophy. Quantification and representative images of C2C12 myotube diameter (10x; enlarged portion within white box) exposed to AML12-LLC conditioned media for 48 hours. Generation of conditioned media: AML12 (CON); AML12+ LLC transwell co-culture (tLLC); AML12+LLC mixed co-culture (mLLC). Significant differences: **p<0.01, ****p<0.0001.
